# miRNA Influences in NRF2 Pathway Interactions within Cancer Models

**DOI:** 10.1155/2015/143636

**Published:** 2015-08-09

**Authors:** Duncan Ayers, Byron Baron, Therese Hunter

**Affiliations:** ^1^Centre for Molecular Medicine and Biobanking, University of Malta, Msida MSD 2080, Malta; ^2^Faculty of Medical and Human Sciences, The University of Manchester, Manchester M1 7DN, UK; ^3^Department of Physiology and Biochemistry, Faculty of Medicine and Surgery, University of Malta, Msida MSD 2080, Malta

## Abstract

The NRF2 transcription factor (nuclear factor-erythroid 2 p45-related factor 2) has been identified as a key molecular player in orchestrating adaptive cellular interactions following a wide spectrum of cellular stress conditions that could be either extracellular or intracellular. Dysregulation of the NRF2 system is implicated in various disease states, including inflammatory conditions. The NRF2 transcription factor is also known to permit cross talk with several other essential cellular signaling pathways. Recent literature has also elucidated the potential influences of miRNA activity over modulations of the NRF2 signalling network. Consequently, further delving into the knowledge regarding the extent of miRNA-induced epigenetic gene regulatory control on key elements of the NRF2 signalling pathway and its cross talk, particularly within the context of cancer models, can prove to be of high clinical importance. This is so since such miRNAs, once identified and validated, can be potentially exploited as novel drug targets for emerging translational medicine-based therapies.

## 1. Introduction

The NRF2 transcription factor (nuclear factor-erythroid 2 p45-related factor 2) has been identified as a key molecular player in orchestrating adaptive cellular interactions following a wide spectrum of cellular stress conditions that could be either extracellular or intracellular [1, 2]. In particular, NRF2 is known to affect cellular sensitivity levels for pathological and physiological mechanisms that are highly influenced by electrophilic and oxidative stress sources, including inflammatory and carcinogenesis processes [1, 2]. It does so by binding to the antioxidant response element (ARE) and the electrophile response element EpRE of a large number of cytoprotective gene promoters. Dysregulation of the NRF2 system is implicated in various disease states such as lung cancer, ovarian, prostate, and breast cancer and inflammatory conditions including hepatitis, diabetes, atherosclerosis, and neurodegenerative disease.

In order to achieve its signaling roles, the NRF2 transcription factor is well known to interact highly with proteins such as KEAP1 (Kelch-like ECH-associated protein 1) [3], though the exact nature of how these interactions lead to the exact pathways for sensing and transducing chemical signaling from varied stress stimuli is still in the course of being elucidated [2]. Recent studies have demonstrated that KEAP1 is highly effective in regulating NRF2 expression through its binding with the latter [4, 5]. This regulation is attained by homeostatic ubiquitination and elimination of NRF2 during cellular activity without the presence of specific stress conditions [5]. However, this KEAP1-induced regulatory mechanism for NRF2 is temporarily halted during time periods where stress triggered circumstances manifest themselves within the cell(s) [5].

**Figure 1:**
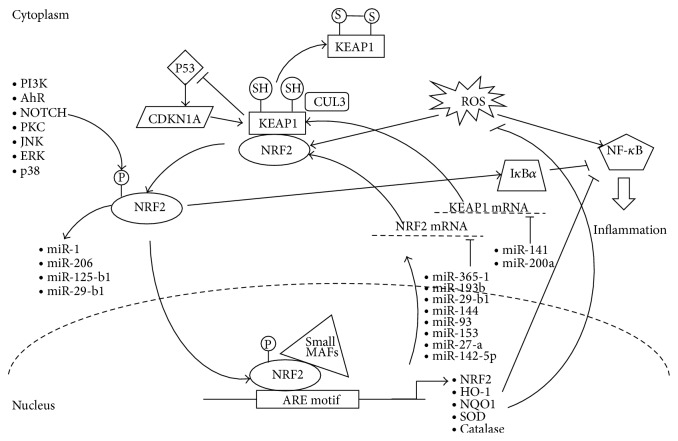
Known regulatory mechanisms of NRF2, highlighting miRNA-mediated influences. Important regulatory pathways include the inhibition of the NF-*κ*B proinflammatory and ROS pathways. Eight miRNAs have also been identified as direct modulators of NRF2 expression at the transcriptomic level.

Furthermore, the NRF2 transcription factor is known to permit cross talk with several other essential cellular signaling pathways (see Figure 1). These include the aryl hydrocarbon receptor (AhR, involved in minimizing xenobiotic toxic activities), the nuclear factor *κ*-light-chain-enhancer of activated B cells (NF-*κ*B) pathway, the p53 pathway (involved in over 50% of human cancers), and the NOTCH signaling pathway [6–9]. Such cross talk interactions can occur through several molecular processes, typical examples being posttranslational modification such as phosphorylation and cysteine redox regulation of target molecular players within the downstream signaling pathway/s concerned, ultimately leading to gene regulation. In turn, cellular activities are affected heavily through such cross talk, leading to actual variations in multiple cellular phenotypes, including the inhibition of cytokine-mediated inflammatory processes and affecting the cellular capacity to remove cellular toxins through the use of drug efflux transporters [2].

Ultimately, it can be inferred that these NRF2 cross talks with other signaling pathways are of clinical importance within the context of many human disease condition pathogeneses, particularly those with highly complex multifactorial molecular interactions, as in most cancer conditions. Consequently, it is crucial to investigate in more detail the exact nature of the NRF2 molecular cross talk, both at the transcript and protein levels, in order to highlight novel molecular players that can eventually be recognized as reliable drug targets in the course of developing novel translational medicine-based molecular therapies for a spectrum of human disease condition, including cancer.

**Figure 2:**
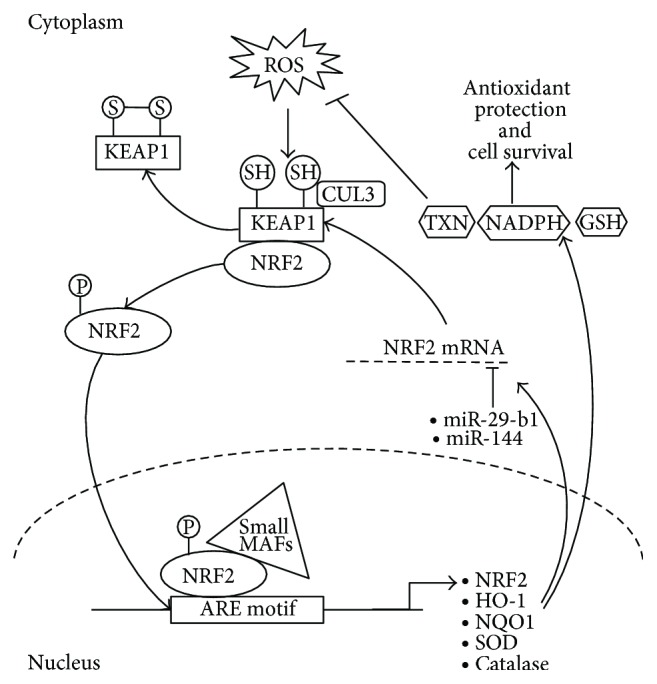
Overview of ROS-mediated NRF2 activities. A feedback mechanism exists, which provides a fine balance between free circulating cytoplasmic NRF2 and nuclear ARE motif-bound NRF2, with miRNAs such as miR-29-b1 and miR-144 providing direct regulation of NRF2 expression within the cytoplasm.

The complexity of the NRF2 system lies in the manner in which it is regulated by the cellular redox environment. Electrophilic/oxidative stress regulates its expression, its interaction with coactivators, its targeted degradation via the proteasome system, the induction of target cytoprotective genes, and its modulation of cellular physiology by interaction with components of other pathways, including miRNA influences, particularly in cancer conditions (see Figure 2). For this reason the system will be studied at the level of DNA, RNA, and protein, both under in vivo and in vitro conditions.

The first ever documented discovery of the existence of microRNAs (miRNAs) dates back to the early 1990s, where researchers focusing on the development of the* C. elegans* nematode identified the gene lin-4 that allowed for the production of small noncoding RNA products that affected growth at the larval stage [10]. Following this revolutionary scientific revelation leading to the ever developing field of miRNA discovery, there are now over 2000 miRNA sequences identified in humans alone (nomenclatured with the prefix hsa-), whereby sequences are catalogued in a comprehensive, publically accessible database known as miRBase (http://www.mirbase.org/) [11]. Throughout the last decade, ever more research has revealed the link between miRNA dysregulations and cancer conditions [12–16]. The key molecular roles of miRNAs in tumourigenesis can be twofold. The first instance is when a tumour-inducing miRNA exhibits upregulated expression within the tumour [17]. The second instance is when a specific tumour suppressor miRNA exhibits downregulated expression in the tumour [17].

**Figure 3:**
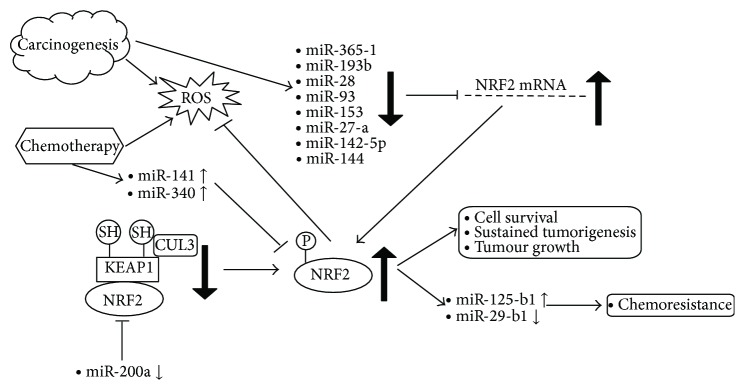
Overview of carcinogenesis and NRF2 responses. Tumour progression is recognized to induce dysregulated miRNA expression, resulting in exacerbated NRF2 activity. Such downstream effects include reduced apoptotic mechanism induction and exacerbation of chemoresistance properties by the tumour, therefore thwarting any conventional cancer therapeutic success.

Recent literature has also elucidated the potential influences of miRNA activity over modulations of the NRF2 signalling network [18, 19]. These modulations can be described either as miRNAs involved in the regulation of NRF2 activity (affectors) or as miRNA mediators of NRF2 activity (effectors) (see Figure 3). This review serves to summarise the seminal research carried out in recent years to identify and elaborate on the varying interactions between miRNAs and the NRF2/Keap1 pathways.

## 2. Affector miRNAs on NRF2 Activity

Chorley and colleagues focused on the utilization of chromatin immunoprecipitation DNA deep sequencing (ChIP-Seq) from lymphoblastoid cell lines, prior to and after controlled exposure periods to oxidative stress, in order to identify potential downstream genes and other molecular players associated with oxidative stress-induced NRF2 activity [20]. The results of the ChIP-Seq screens, using an antibody for NQO1 ARE, elucidated peak regions corresponding to the locations of multiple miRNAs, with the most notable being the miR-365-1/miR-193b cluster and miR-29b-1 [20]. The importance of these findings was that the miR-365-1/miR-193b cluster was recognized to be dysregulated in multiple cancer conditions, while miR-29b-1 was linked directly to oxidative stress in a previous study [20, 21].

Another study recognized the functional link between miR-144 and NRF2 activity during periods of oxidative stress [22]. The study by Sangokoya and colleagues identified the upregulated expression of miR-144 from erythrocyte samples of homozygous sickle cell disease (HbSS) patients suffering from exacerbated anaemic states [22]. Consequently, luciferase assays confirmed the direct regulatory effect of miR-144 on NRF2 expression, with the latter bearing two target sequences for miR-144 on the 3′-UTR for NRF2 [22]. The results of this study suggest that miR-144 has a major role in controlling the oxidative stress regulatory system for erythrocytes in patients affected by sickle cell disease [22].

Similarly, the study performed by Yang and colleagues revealed the direct regulatory effect of miR-28 on NRF2 expression and consequent cellular functions in breast carcinoma cell lines [23]. Luciferase reporter assays confirmed that miR-28 successfully regulated NRF2 expression through binding on the NRF2 3′-UTR, with this link being further validated at both the transcriptomic and proteomic levels [23]. However, it was also elucidated that the regulatory effect of miR-28 is independent of Keap-1, since overexpression of miR-28 did not induce any dysregulated expression of Keap-1 nor did it affect Keap-1 and NRF2 interactions [23].

Singh and colleagues highlighted the regulatory role of miR-93 on NRF2 activity within rat models of breast carcinogenesis [24]. In this case, the study had elucidated a reduction in NRF2 protein levels following induced expression of miR-93, together with a reduction in carcinogenesis-associated phenotypes (mammosphere development, antiapoptosis, and DNA damage) following miR-93 knockdown assays [24].

Neuroblastoma cell line models also exhibited miRNA-regulated NRF2 activities, as demonstrated by Narasimhan and colleagues [25]. The study identified miR-153, miR-27-a, miR-142-5p, and miR-144 as regulatory miRNAs for NRF2 within the SH-SY5Y neuroblastoma cell line [25]. Such regulatory links were validated through cotransfection assays using individual miRNA mimics, together with the result of reduced NRF2 transcript and protein levels following ectopic expression of the miRNAs [25]. This suggests that the miRNA regulatory role in this situation is direct, with no dependence on Keap1 for successful modulation of NRF2 expression [25].

## 3. NRF2-Driven Effector miRNAs

Singh and colleagues elucidated the tumourigenic activity exhibited by NRF2 through regulating miR-1 and miR-206 activity. Gain of function for NRF2 expression in A549 cells revealed a consequent downregulated expression level for both miRNAs with similar downregulatory effects on downstream genes that are primarily involved in the pentose phosphate pathway (PPP) [26]. Ultimately, the gain of NRF2 function allowed for enhanced glucose metabolism within such tumour cells.

The NRF2 transcription factor is also found to be upregulated in acute myeloid leukaemia (AML). Consequently, Shah and colleagues investigated the possible miRNA dysregulations occurring due to NRF2 upregulation within AML [27]. The results of miRNA microarray screenings performed after NRF2 knockdown in AML cells highlighted NRF2-directed upregulated expression of miR-125-b1 and concomitant downregulated expression of miR-29-b1 [27]. Transient transfection assays using miR-125-b1 antagomiR and miR-29-b1 mimics consequently confirmed their role in the prevention of apoptosis within AML cell populations [27].

Further evidence for the transcriptional control of NRF2 on miR-125b1 expression was presented through the study conducted by Joo and colleagues [28]. This study highlighted the increase in miR-125-b1 expression following activation of NRF2 within kidney tissues of mice treated with oltipraz [28]. The study also revealed that miR-125-b1 expression allows for chemoprotection of murine kidney cells from cisplatin cytotoxicity [28].

## 4. NRF2/miRNA Interplays and Cancer Chemoresistance

The influence of miRNA-NRF2 interplays can also affect other tumour characteristics, apart from tumourigenesis. An example of such activity is the chemosensitivity properties of tumours to individual and/or multiple chemotherapeutic agents, also known as multidrug resistance (MDR).

The phenomenon of MDR within a tumour can be either innate (due to the inherent genetic composition of the tumour) or acquired through selection following multiple exposure periods to chemotherapy [17, 29]. Furthermore, there are multiple pathways through which a tumour can acquire MDR properties, with the most common pathway being the upregulated expression of drug efflux pump genes such as the ABC transporter genes [30].

Shi and colleagues revealed an upregulated expression of miR-141 that correlated with drug resistance to 5-fluorouracil within hepatocellular carcinoma (HCC) cell line chemoresistance models for the drug [31]. Further analysis using real time quantitative polymerase chain reaction (RT-qPCR) and luciferase reporter assays revealed that miR-141 exhibited a direct regulatory effect on the downstream Keap1 transcript [31]. In addition, exacerbation of miR-141 function through transient transfection of miR-141 mimics allowed for increased chemoresistance to 5-fluorouracil by HCC cell lines [31]. Another study conducted by the same group similarly revealed the direct regulatory effect of miR-340 on NRF2 within HCC cell lines bearing a chemoresistance phenotype for cisplatin [32]. The study elucidated that upregulation of miR-340 directly affected NRF2 transcript expression, with a consequent result of reduction in the level of cisplatin chemoresistance properties by the cell lines analysed in this study [32].

The study by Joo and colleagues serves as an additional example of NRF2 controlled upregulation of miRNAs (miR-125-b1 in this case) as a means of exacerbating chemoresistance against specific cytotoxic drugs (cisplatin in this case) routinely used in cancer chemotherapy [28].

Another study conducted by Murray-Stewart and colleagues identified miR-200-a as a mediating agent for aiding naturally occurring polyamine analogue-based histone deacetylase inhibitors in reverting chemoresistance properties within non-small-cell lung carcinoma cell lines [33]. The study concluded that the polyamine analogue-induced miR-200-a expression allowed for targeted regulation of Keap1 transcript expression, resulting in NRF2 binding to spermidine/spermine *N* ^1^-acetyltransferase (SSAT) promoter regions [33]. Since SSAT is a polyamine catabolic enzyme that plays a major role in chemosensitivity to antitumour drugs, this miRNA/NRF2 interplay leads to lowering the intracellular levels of polyamines within tumour cells and consequently imposing a negative effect on tumour growth [33]. Similar evidence for the interplay between miR-200-a and NRF2 was highlighted through the study performed by Eades and colleagues in breast carcinoma cell lines [34]. This study also confirmed that miR-200-a binds successfully to the 3′-UTR of the Keap1 transcript, resulting in direct regulation posttranscriptionally [34].

## 5. NRF2/miRNA Interplays and Other Cancer Characteristics

The molecular interactions and regulatory mechanisms occurring between NRF2 and components of the miRnome are not just limited to tumour chemoresistance properties. Tumour proliferation and metastasis properties can also be regulated through such interplays. Cortez and colleagues recognized the influences of miR-200c on non-small-cell lung carcinoma cell lines [35]. The results of this study, following miR-200c overexpression, demonstrated the effects of miR-200c on enhancing cell line radiosensitivity through increased apoptotic triggering [35]. In addition, the study also highlighted that miR-200c overexpression had a direct regulatory effect on oxidative stress responses, primarily GABP/NRF2 and SESN1 expression [35].

Hypoxia-induced interactions can also affect Nrf2 expression through indirect regulation by miRNAs [36]. Hypoxia-inducible factor 1*α* (HIF-1*α*) is recognized to downregulate NRF2 expression and also affect expression of several miRNAs, including the oncomiR polycistron of miR-17-92 [36]. Furthermore, the hypoxic state itself can also possibly induce specific miRNA dysregulations leading to the biased expression of HIF-1*α* instead of HIF-2*α*, where the latter does not have any regulatory effects on NRF2 expression [36].

Tumourigenesis can also be influenced through NRF2 and miRNA interplays, as identified in the study by Singh and colleagues [26]. Knockdown NRF2 within A549 tumour cell lines exhibited a reduction in cell line growth and concomitantly downregulated the expression of two miRNAs, miR-1 and miR-206, respectively [26]. The results of this study also elucidated that such NRF2-directed miRNA modulations allow for the enhanced flow of carbon to the tricarboxylic acid cycle and pentose phosphate pathway, leading to modifications in glucose metabolism and ultimately exacerbating tumour cell growth and proliferation [26].

## 6. NRF2/miRNA Interplays with Other Molecular Pathways

The molecular interplays occurring between NRF2 and other downstream molecular pathways can also be mediated by NF2-driven miRNA modulations. A typical example for this being the study by Joo and colleagues [28]. The results of this study confirmed the upregulation of miR-125b through NRF2 expression, which in turn regulated the expression of aryl hydrocarbon receptor repressor (AhRR), resulting in reduction in regulation of cellular growth within murine renal tissues exposed to cisplatin [28]. Furthermore, the study also revealed the indirect regulation of cisplatin-induced p53 activation by miR-125-b through the exacerbated expression of MDM2 as a downstream effect of increased aryl hydrocarbon receptor (AhR) cellular levels [28].

In a similar manner, the interactions between NRF2 and the proinflammatory NF-*κ*B pathways can be affected by miRNA activity [37]. The study by Wagner and colleagues illustrated the downregulated expression of miR-155 following allyl-isothiocyanate in murine RAW 264.7 macrophage cell lines [37]. This miRNA downregulated expression was also correlated to increased NRF2 expression and a severe reduction in the expression of the proinflammatory tumour necrosis factor *α* gene [37].

## 7. Conclusions

In essence, this paper serves to highlight the clinical importance of miRNA influences within the NRF2/KEAP1 pathway and other significant pathways that are in turn affected by NRF2 activity.

The importance of elucidating such novel molecular interplays at the miRnomic level cannot be emphasized enough, since any dysregulated miRNA deemed to be involved in contributing to exacerbation of specific tumour characteristics and clinical progression can be identified as a novel drug target. In addition, the identification of such miRNA dysregulated expression profiles within tumour biopsy samples during patient diagnosis will allow clinicians to base any clinical treatments according to the enhanced picture regarding the exact tumour characteristics within the individual patient, therefore allowing for more effective and bespoke therapies and ultimately increasing the possibility of a successful prognosis for the patient.

Further delving into the knowledge regarding the extent of redox sensitive miRNA-induced epigenetic gene regulatory control on key elements of the NRF2 signalling pathway and its cross talk, particularly within the context of cancer models, can prove to be of high clinical importance. This is so since such miRNAs, once identified and validated, can be potentially exploited as novel drug targets for emerging translational medicine-based therapies within clinical settings in the near future.
